# Emerging Enterovirus A71 Subgenogroup B5 Causing Severe Hand, Foot, and Mouth Disease, Vietnam, 2023

**DOI:** 10.3201/eid3002.231024

**Published:** 2024-02

**Authors:** Nguyen Van Vinh Chau, Tang Chi Thuong, Nguyen Thanh Hung, Nguyen Thi Thu Hong, Du Tuan Quy, Tran Ba Thien, Cao Minh Hiep, Ngo Ngoc Quang Minh, Truong Huu Khanh, Do Duong Kim Han, Truong Hoang Chau Truc, Nguyen Thi Han Ny, Le Kim Thanh, Lam Anh Nguyet, Cao Thu Thuy, Le Nguyen Truc Nhu, Pham Van Quang, Phung Nguyen The Nguyen, Phan Tu Qui, H. Rogier van Doorn, C. Louise Thwaites, Tran Tan Thanh, Nguyen Thanh Dung, Guy Thwaites, Nguyen To Anh, Le Nguyen Thanh Nhan, Le Van Tan, for the SEACOVARIANTS

**Affiliations:** Department of Health, Ho Chi Minh City, Vietnam (N.V.V. Chau, T.C. Thuong);; Children’s Hospital 1, Ho Chi Minh City (N.T. Hung, D.T. Quy, C.M. Hiep, N.N.Q. Minh, T.H. Khanh, P.V. Quang, P.N.T. Nguyen, L.N.T. Nhan);; Oxford University Clinical Research Unit, Ho Chi Minh City (N.T.T. Hong, T.B. Thien, D.D.K. Han, T.H.C. Truc, N.T.H. Ny, L.K. Thanh, L.A. Nguyet, C.T. Thuy, L.N.T. Nhu, H.R. van Doorn, C.L. Thwaites, T.T. Thanh, G. Thwaites, N.T. Anh, L.V. Tan);; Pham Ngoc Thach University of Medicine, Ho Chi Minh City (P.V. Quang);; University of Medicine and Pharmacy at Ho Chi Minh City, Ho Chi Minh City (P.N.T. Nguyen);; Hospital for Tropical Diseases, Ho Chi Minh City (P.T. Qui, N.T. Dung);; University of Oxford, Oxford, UK (H.R. van Doorn, C.L. Thwaites, G. Thwaites, L.V. Tan)

**Keywords:** enteroviruses, enterovirus A71, subgenogroup B5, hand foot and mouth disease, outbreaks, emerging infections, enteric infections, viruses, Vietnam

## Abstract

We report on a 2023 outbreak of severe hand, foot, and mouth disease in southern Vietnam caused by an emerging lineage of enterovirus A71 subgenogroup B5. Affected children were significantly older than those reported during previous outbreaks. The virus should be closely monitored to assess its potential for global dispersal.

Since 1997, large outbreaks of severe hand, foot, and mouth disease (HFMD) caused by diverse enterovirus A71 (EV-A71) subgenogroups (such as B4, B5, C4, and C5) have been reported in the Asia Pacific region (*1*), resulting in millions of hospitalizations and substantial numbers of deaths. Increased EV-A71 detection and associated neurologic disease have also been documented worldwide, including in the United States in more recent years (*2*).

During January 1–June 30, 2023, a total of 12,600 HFMD cases and 7 deaths were reported in Vietnam. Of those cases, 5,383 (42.7%) infections and all 7 deaths were recorded in June 2023. We investigated the epidemiologic and virologic features of this outbreak. The study was approved by the Institutional Review Board of CH1 and the Oxford University Tropical Research Ethics Committee. Written informed consent was obtained from a parent or guardian of each enrolled patient.

## The Study

This study forms part of an ongoing HFMD research program conducted at Children’s Hospital 1 (CH1) in Ho Chi Minh City, Vietnam, since 2013 (Appendix) (*3*). Recruited patients had clinical data recorded and throat and rectal swab samples collected for virologic investigation of EV-A71 and other enterovirus infections (Appendix Figure 1) (*4*,*5*). We extracted complementary data from hospital records or from a clinical study conducted during 2013–2018 (*3*). 

We generated EV-A71 whole-genome sequences directly from virus-positive rectal or throat swab samples that had sufficient viral loads (PCR cycle threshold values of <30) by using a metagenomics-based approach, as previously described (*6*). We performed recombination analysis by using the Chimera, GENECONV, Maxchi, Bootscan, and Siscan algorithms available in RDP4 software (*7*). To assess virus evolution, we constructed maximum-likelihood phylogenetic trees for enterovirus viral protein 1 (VP1) and whole-genome sequences by using IQ-TREE (*8*); we obtained representative global sequences from GenBank for comparisons (Appendix Tables 1, 2). 

**Figure 1:**
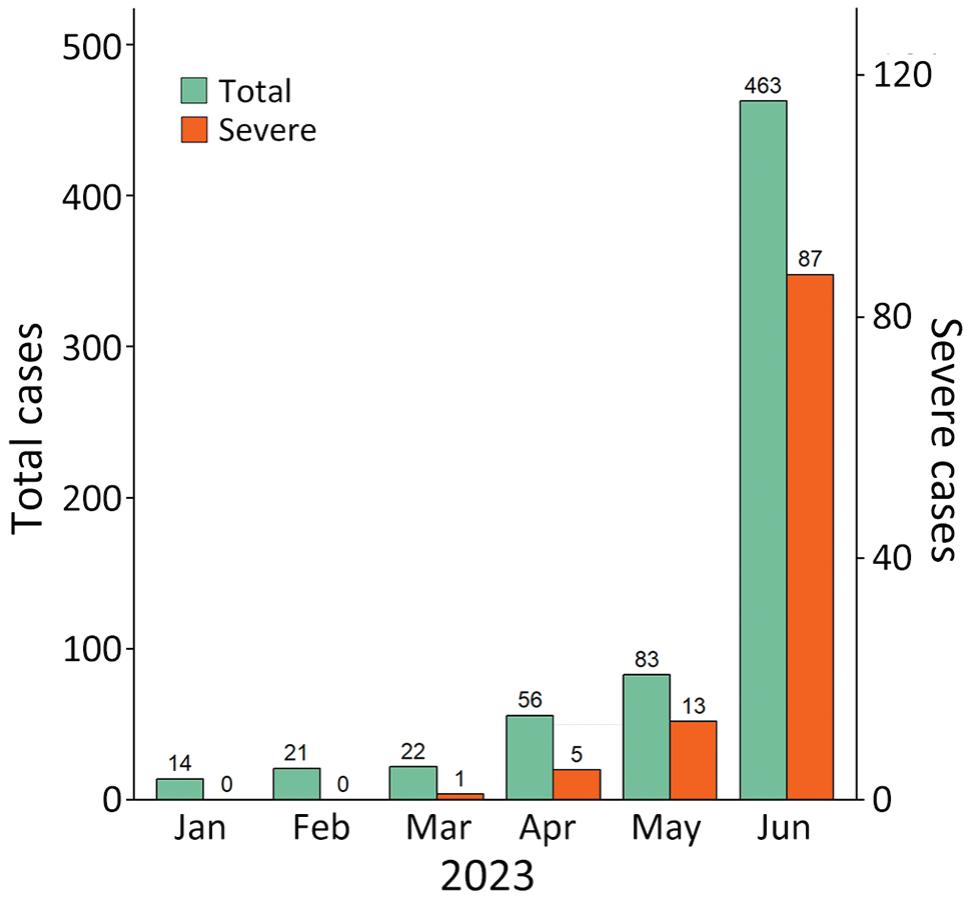
Admissions for and severe cases of hand, foot, and mouth disease recorded during January–June 2023 at Children’s Hospital 1, Ho Chi Minh City, Vietnam, in study of emerging enterovirus A71 subgenogroup B5. Green bars indicate total number of patients admitted for hand, foot, and mouth disease. Red bars indicate the number of admitted patients who had severe disease. Numbers above bars indicate actual number of cases at each time point. Scales for the y-axes differ substantially to underscore patterns but do not permit direct comparisons.

During January–June 2023, a total of 659 children with HFMD (including 106 with severe cases) were admitted to CH1; most admissions (463/659 [70.3%]) and severe cases (87/106 [82.1%]) occurred in June (Figure 1). Of the 659 children, 101 participated in this study. The participants resided in 15 provinces/cities in southern Vietnam (Appendix Figure 2) and were admitted to CH1 shortly after illness onset; the median number of illness days before admission was 2 (interquartile range 1–2) (Table 1). Twenty-eight (27.7%) participants had a disease severity grade of 2A, and 73 (72.3%) had grade 2B1 or worse (Table 1). Disease progressed from lower to higher severity grade in 63 (62.4%) of 101 children; clinical manifestations progressed within 1 day after admission in 47 (74.6%) children (Appendix Figure 3).

**Table 1:** Demographics of patients with enterovirus infections and clinical grades of hand, foot, and mouth disease in study of emerging EV-A71 subgenogroup B5, Vietnam, 2023*

Characteristics	Total, n = 101	EV-A71, n = 83	EV-A71 B5, n = 65	PCR negative, n = 17
Sex				
M	61 (60.4)	48 (57.8)	39 (60.0)	12 (70.6)
F	40 (39.6)	35 (42.2)	26 (40.0)	5 (29.4)
Median age, mo (IQR)	26 (19–34)	27 (21–36)	28 (21–36)	20 (15–22)
Illness at admission, median d (IQR)†	2 (1–2)	2 (1–2)	2 (1–3)	2 (1–2)
Origin of patients				
Ho Chi Minh City	46 (45.5)	35 (42.2)	28 (43.1)	10 (58.8)
Other provinces/cities	55 (54.5)	48 (57.8)	37 (56.9)	7 (41.2)
Clinical grade of disease‡				
2A	28 (27.7)	17 (20.5)	15 (23.1)	10 (58.8)
2B1	15 (14.9)	12 (14.5)	11 (16.9)	3 (17.6)
2B2	16 (15.8)	15 (18.1)	12 (18.5)	1 (5.9)
3	41 (40.6)	39 (47.0)	27 (41.5)	2 (11.8)
4	1 (1.0)	0 (0.0)	0 (0.0)	1 (5.9)

*Values are no. (%) except as indicated. EV-A71, enterovirus A71; IQR, interquartile range. †Median number of days of illness before admission. ‡Clinical grades of hand, foot, and mouth disease have been previously defined (*3*).

We detected enteroviruses in samples from 84 (83.2%) of 101 patients. Of those 84 patients, 83 (98.8%) were positive for EV-A71, and 1 patient was positive for coxsackievirus A5. We determined the subgenogroup for 67 samples and assigned 65 samples to subgenogroup B5 (Table 1) and 2 samples to subgenogroup C1. The 2 C1-infected patients had grade 2B1 and grade 3 disease severity. Compared with EV-A71–infected children enrolled in the clinical study during 2013–2018, those in the 2023 outbreak were significantly older (Table 2; Appendix Figure 4).

**Table 2:** Age comparisons among patient groups infected with different enterovirus subgenogroups over time in study of emerging EV-A71 subgenogroup B5 causing severe hand, foot, and mouth disease, Vietnam, 2023*

Age	EV-A71				EV-A71 B5				EV-A71 C4†	
	2023	2013–2018	p value		2023	2013–2018	p value		2013–2018	p value
Median age, mo (IQR)	27 (21–36)	21 (15–31)	<0.001		28 (21–36)	18 (13–30)	<0.001		22 (17–33)	0.042

*Wilcoxon rank-sum test with continuity correction was applied for analyses of patient ages among groups. EV-A71, enterovirus A71; IQR, interquartile range. †Comparison was between EV-A71 subgenogroup B5 detected during 2023 vs. EV-A71 subgenogroup C4 detected during 2013–2018.

**Figure 2:**
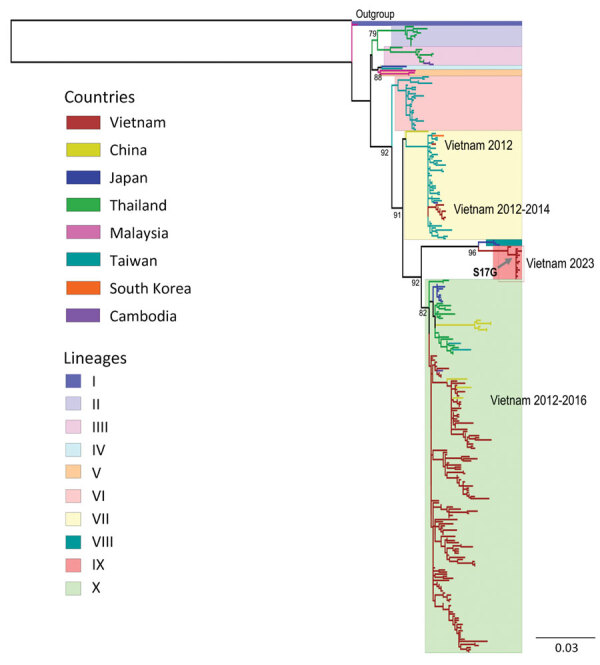
Phylogenetic analysis of viral protein 1 (VP1) coding sequences in study of emerging enterovirus A71 subgenogroup B5 causing severe hand, foot, and mouth disease, Vietnam, 2023. Tree was constructed for VP1 gene sequences by using the maximum-likelihood method to compare genetic relatedness among the B5 sequences from this study and global sequences obtained from GenBank. Line colors indicate the country of origin for each sequence. Box colors indicate the enterovirus lineage. Arrow indicates the emerging B5 lineage from Vietnam carrying an S17G codon substitution within the N-terminus of VP1. Similar phylogenetic tree structure was obtained when the analysis was performed by using complete genome coding sequences. Interlineage and intralineage nucleotide sequence similarities among the lineages were calculated (Appendix Table 3). Scale bar indicates nucleotide substitutions per site.

We obtained whole-genome sequences from 16 B5-positive samples (14 rectal and 2 throat swab samples from 16 individual patients) (Appendix Table 2). We did not detect recombination events. Phylogenetic analysis indicated the B5 viruses in Vietnam were most closely related to the B5 viruses from Japan, but they formed a distinct lineage from those previously isolated from Vietnam and worldwide (Figure 2; Appendix Table 3, Figure 5). In addition, 15 of 16 B5 sequences from the 2023 outbreak carried a glycine residue at position 17 (G17) within the N-terminus of VP1. In the 1 remaining sample, a G17 codon was detected in 3 of 122 reads generated by the metagenomic workflow, and a serine (S17) codon was detected in the remaining 119 reads (Appendix Figure 6). In contrast, among 287 nonidentical global B5 sequences used for phylogenetic analysis, an S17 codon was observed in 285 (99.3%) and a G17 codon was observed in 2 (0.7%) sequences. However, the 2 G17-containing sequences were derived from virus isolates passaged in cultured cell lines (*9*). Because of the small number of subgenogroup C1 sequences (n = 2), we deemed a similar in-depth analysis to be uninformative, but the C1 viruses from this study were closely related phylogenetically to C1 strains isolated worldwide (Appendix Figure 7).

## Conclusions

We report that the 2023 outbreak of severe HFMD in Vietnam was caused by EV-A71 subgenogroups B5 and C1; B5 is dominant, and more older children were affected than during previous outbreaks. Phylogenetic analyses suggest that both B5 and C1 viruses were derived from new introductions of EV-A71 into Vietnam. In addition, the B5 viruses likely represent an emerging lineage because of a unique nonsynonymous amino acid substitution (S17G) in VP1 and because they form a distinct lineage within the global B5 phylogenetic tree. Further research is needed to clarify the origin and transmission network of this emerging lineage.

Underlying factors might cause the emergence of EV-A71 subgenogroups within a specific locality; the accumulation of a sufficient number of susceptible young children in the population and pathogen evolution might play critical roles (*9*,*10*). The changing epidemiology of respiratory pathogens as a consequence of COVID-19 has been documented (*11*), although EV-A71 is mainly transmitted by the oral-fecal route; thus, the effects of COVID-19 on EV-A71 transmission might be different from those of other respiratory viruses. However, the COVID-19 pandemic could have resulted in a large cohort of children who had greater susceptibility to EV-A71 infection, leading to a surge in infections among older children in the 2023 outbreak. Virus immune evasion or altered virulence might also be substantial contributing factors in the outbreak (*9*,*12*). The amino acid residue 17 in VP1 does not form part of the identified EV-A71 immune epitopes (*13*), but mutations in the N terminus of VP1 might increase cell tropism, potentially contributing to EV-A71 pathogenesis. Collectively, because VP1 is the most immunogenic protein of EV-A71, the potential effects of the nonsynonymous S17G substitution on immune escape and virulence of EV-A71 subgenogroup B5 warrant further investigation.

Previous peaks of EV-A71 outbreaks in Vietnam occurred during September–November (*3*), coinciding with school reopening after the summer holiday (June–August). As of November 2023, the outbreak in Vietnam was still ongoing and had resulted in >100,000 infections and 23 deaths across the country. The potential for severe EV-A71–associated HFMD outbreaks to spread to other parts of the world should be closely monitored.

Inactivated EV-A71 vaccines have been developed in China and Taiwan (*14*) but have only been used in China. Real-world data have shown that those vaccines substantially reduced EV-A71–associated disease transmission in China (*15*). Thus, using EV-A71 vaccines in other HFMD-endemic countries could have a similar effect. However, the extent to which EV-A71 vaccines might shape HFMD dynamics as a whole should be closely monitored. Because HFMD is transmitted through the oral-fecal route, good hygiene is critical to reduce EV-A71 transmission. 

In conclusion, the 2023 outbreak of severe HFMD in Vietnam has mainly been caused by an emerging EV-A71 subgenogroup B5 lineage, and older children have been affected. Clinicians should recognize the diverse clinical manifestations of HFMD. Furthermore, enhanced EV-A71 surveillance is needed to inform the outbreak response in Vietnam and elsewhere, should the virus spread.

Appendix Additional information for emerging enterovirus A71 subgenogroup B5 causing severe hand, foot, and mouth disease, Vietnam, 2023.
