# Supplementary material for: Emerging Enterovirus A71 Subgenogroup B5 Causing Severe Hand, Foot, and Mouth Disease, Vietnam, 2023
Source: Emerg Infect Dis. 2024 Feb;30(2):363–7. doi: 10.3201/eid3002.231024 (PMC10826755; doi:10.3201/eid3002.231024)
Supplement: Appendix — Additional information for emerging enterovirus A71 subgenogroup B5 causing severe hand, foot, and mouth disease, Vietnam, 2023. [file 23-1024-Techapp-s1.pdf]

Article DOI: https://doi.org/10.3201/eid3002.231024
EID cannot ensure accessibility for supplementary materials supplied by authors. Readers who have difficulty accessing supplementary content should contact the authors for assistance.

Emerging Enterovirus A71 Subgenogroup B5 Causing Severe Hand, Foot, and Mouth Disease, Vietnam, 2023

Appendix

Additional Methods

Hand, Foot, and Mouth Disease Grade Classification

The Vietnam Ministry of Health subdivides HFMD into 4 major clinical grades. Grade 1 is assigned to patients with mouth ulcers or vesicles/papules on their hands, feet, or buttocks, with or without mild fever (<39°C). Grade 2 is further divided into grade 2A (central nervous system involvement: myoclonus reported by parents or caregivers only, fever >39°C, or ataxia); grade 2B1 (myoclonus observed by medical staff or history of myoclonus and lethargy or pulse >130 beats per minute); and grade 2B2 (ataxia, cranial nerve palsies, limb weakness, nystagmus, persistent high fever, or pulse >150 beats per minute). Grade 3 involves autonomic dysfunction, such as profuse sweating, hypertension, tachycardia, and tachypnea, and grade 4 is assigned for disease with additional cardiopulmonary compromise, such as pulmonary edema or shock syndrome. Patients with clinical grade ≥2B1 are considered to have severe HFMD and require close monitoring.

Study and Setting

Children's Hospital 1 in Ho Chi Minh City, Vietnam, is a 1,600-bed pediatric hospital and 1 of 3 tertiary referral centers for children with hand, foot, and mouth disease (HFMD) in southern Vietnam, which has a population of >40 million persons. Study recruitment focused on hospitalized patients with HFMD; ≈50% of patients had a clinical disease grade of 2A or 2B1 and ≈50% had a grade of ≥2B2. During 2019–April 2023, recruitment to the study was interrupted because of low case numbers of HFMD and the emergence of COVID-19.

Enterovirus Diagnostics and Serotype Determination

Enterovirus infection diagnosis and serotyping/genogrouping were performed by using a combination of PCR and sequencing approaches (Appendix Figure 1) (*1–3*). In brief, we extracted virus RNA from rectal swab samples collected from study participants and then used one-step multiplex real-time reverse transcription PCR (RT-PCR) to simultaneously detect enteroviruses and enterovirus A71 (EV-A71). Any specimens positive for enteroviruses or EV-A71 were then tested further to identify specific enterovirus serotypes or EV-A71 subgenogroups by using a combination of PCR amplification and sequencing of the viral protein 1 gene amplicon (*2*,*3*). Viral protein 1 sequences were then analyzed by using a previously described online tool to determine enterovirus serotype or EV-A71 subgenogroup (*4*). If the RT-PCR analysis of a rectal sample was negative, throat swab samples were analyzed, and, if positive, the same subsequent steps were repeated to identify enterovirus serotypes or EV-A71 subgenogroups. A confirmed enterovirus diagnosis was established if either a throat swab or rectal swab sample was positive by real time RT-PCR.

References

1. Thanh TT, Anh NT, Tham NT, Van HMT, Sabanathan S, Qui PT, et al. Validation and utilization of an internally controlled multiplex real-time RT-PCR assay for simultaneous detection of enteroviruses and enterovirus A71 associated with hand foot and mouth disease. Virol J. 2015;12:85. PubMed https://doi.org/10.1186/s12985-015-0316-2

2. Nix WA, Oberste MS, Pallansch MA. Sensitive, seminested PCR amplification of VP1 sequences for direct identification of all enterovirus serotypes from original clinical specimens. J Clin Microbiol. 2006;44:2698–704. PubMed https://doi.org/10.1128/JCM.00542-06

3. Tan le V, Tuyen NTK, Thanh TT, Ngan TT, Van HMT, Sabanathan S, et al. A generic assay for whole-genome amplification and deep sequencing of enterovirus A71. J Virol Methods. 2015;215–216:30–6. PubMed https://doi.org/10.1016/j.jviromet.2015.02.011

4. Kroneman A, Vennema H, Deforche K, v d Avoort H, Peñaranda S, Oberste MS, et al. An automated genotyping tool for enteroviruses and noroviruses. J Clin Virol. 2011;51:121–5. PubMed https://doi.org/10.1016/j.jcv.2011.03.006

Appendix Table 1. Accession numbers and geographic origins of enterovirus A71 sequences retrieved from GenBank for phylogenetic analyses

Accession no.	Country	Virus
MF662693.1	China	EV-A71 C4
KT354870.1	Taiwan	EV-A71 B5
KT354868.1	Taiwan	EV-A71 B5
KT354869.1	Taiwan	EV-A71 B5
KT354867.1	Taiwan	EV-A71 B5
KT354866.1	Taiwan	EV-A71 B5
MG756708.1	Taiwan	EV-A71 B5
MG756714.1	Taiwan	EV-A71 B5
KF974788.1	Taiwan	EV-A71 B5
KF974780.1	Taiwan	EV-A71 B5
KF974785.1	Taiwan	EV-A71 B5
MG756706.1	Taiwan	EV-A71 B5
KF974783.1	Taiwan	EV-A71 B5
MG756711.1	Taiwan	EV-A71 B5
KF974781.1	Taiwan	EV-A71 B5
KF974787.1	Taiwan	EV-A71 B5
KF974784.1	Taiwan	EV-A71 B5
KF974779.1	Taiwan	EV-A71 B5
MG756710.1	Taiwan	EV-A71 B5
MG756709.1	Taiwan	EV-A71 B5
MG756712.1	Taiwan	EV-A71 B5
MG756707.1	Taiwan	EV-A71 B5
HM156065.1	Taiwan	EV-A71 B5
KF974786.1	Taiwan	EV-A71 B5
FJ357385.1	Taiwan	EV-A71 B5
MG756713.1	Taiwan	EV-A71 B5
HM622390.1	Taiwan	EV-A71 B5
JN964686.1	China	EV-A71 B5
MN629889.1	South Korea	EV-A71 B5
KF154354.2	Taiwan	EV-A71 B5
KF974798.1	Taiwan	EV-A71 B5
MG756731.1	Taiwan	EV-A71 B5
KF974797.1	Taiwan	EV-A71 B5
KF974790.1	Taiwan	EV-A71 B5
MG756723.1	Taiwan	EV-A71 B5
MG756740.1	Taiwan	EV-A71 B5
MG756743.1	Taiwan	EV-A71 B5
MG756729.1	Taiwan	EV-A71 B5
MG756727.1	Taiwan	EV-A71 B5
MG756721.1	Taiwan	EV-A71 B5
KF134486.1	Taiwan	EV-A71 B5
KF974794.1	Taiwan	EV-A71 B5
MG756753.1	Taiwan	EV-A71 B5
MG756735.1	Taiwan	EV-A71 B5
MG756726.1	Taiwan	EV-A71 B5
MG756744.1	Taiwan	EV-A71 B5
KF154355.1	Taiwan	EV-A71 B5
MG756739.1	Taiwan	EV-A71 B5
KJ686137.1	Vietnam	EV-A71 B5
MG756748.1	Taiwan	EV-A71 B5
MG756730.1	Taiwan	EV-A71 B5
KF974791.1	Taiwan	EV-A71 B5
KF974792.1	Taiwan	EV-A71 B5
MG756745.1	Taiwan	EV-A71 B5
MG756732.1	Taiwan	EV-A71 B5
KF974795.1	Taiwan	EV-A71 B5
KF974793.1	Taiwan	EV-A71 B5
MG756751.1	Taiwan	EV-A71 B5
MG756750.1	Taiwan	EV-A71 B5
MG756749.1	Taiwan	EV-A71 B5
MG756747.1	Taiwan	EV-A71 B5
MG756741.1	Taiwan	EV-A71 B5
KF974796.1	Taiwan	EV-A71 B5
MG756752.1	Taiwan	EV-A71 B5

Accession no.	Country	Virus
MG756733.1	Taiwan	EV-A71 B5
MG756754.1	Taiwan	EV-A71 B5
MG756734.1	Taiwan	EV-A71 B5
MG756746.1	Taiwan	EV-A71 B5
MG756742.1	Taiwan	EV-A71 B5
MG756738.1	Taiwan	EV-A71 B5
LC626900.1	Japan	EV-A71 B5
MH716391.1	Vietnam	EV-A71 B5
KJ686176.1	Vietnam	EV-A71 B5
MH716390.1	Vietnam	EV-A71 B5
MH716384.1	Vietnam	EV-A71 B5
MH716382.1	Vietnam	EV-A71 B5
MH716388.1	Vietnam	EV-A71 B5
MH716387.1	Vietnam	EV-A71 B5
MH716392.1	Vietnam	EV-A71 B5
MH716381.1	Vietnam	EV-A71 B5
OM417113.1	Thailand	EV-A71 B5
OM417115.1	Thailand	EV-A71 B5
OM417111.1	Thailand	EV-A71 B5
MG756694.1	Taiwan	EV-A71 B5
KX372331.1	Thailand	EV-A71 B5
KX372316.1	Thailand	EV-A71 B5
KR045300.1	Thailand	EV-A71 B5
KX372315.1	Thailand	EV-A71 B5
KX372314.1	Thailand	EV-A71 B5
KX372325.1	Thailand	EV-A71 B5
KX372326.1	Thailand	EV-A71 B5
KX372323.1	Thailand	EV-A71 B5
LC626875.1	Japan	EV-A71 B5
LC626901.1	Japan	EV-A71 B5
LC626879.1	Japan	EV-A71 B5
LC626878.1	Japan	EV-A71 B5
LC626872.1	Japan	EV-A71 B5
LC626877.1	Japan	EV-A71 B5
LC626876.1	Japan	EV-A71 B5
LC626873.1	Japan	EV-A71 B5
LC626874.1	Japan	EV-A71 B5
KR045299.1	Thailand	EV-A71 B5
MG756695.1	Taiwan	EV-A71 B5
KR045301.1	Thailand	EV-A71 B5
KR045302.1	Thailand	EV-A71 B5
KX372327.1	Thailand	EV-A71 B5
KX372330.1	Thailand	EV-A71 B5
KX372329.1	Thailand	EV-A71 B5
KX430824.1	Vietnam	EV-A71 B5
MH716280.1	Vietnam	EV-A71 B5
MH716279.1	Vietnam	EV-A71 B5
MH716258.1	Vietnam	EV-A71 B5
MH716283.1	Vietnam	EV-A71 B5
MH716281.1	Vietnam	EV-A71 B5
MH716282.1	Vietnam	EV-A71 B5
MH716267.1	Vietnam	EV-A71 B5
MH716272.1	Vietnam	EV-A71 B5
MH716273.1	Vietnam	EV-A71 B5
MH716259.1	Vietnam	EV-A71 B5
MH716264.1	Vietnam	EV-A71 B5
MH716292.1	Vietnam	EV-A71 B5
MH716293.1	Vietnam	EV-A71 B5
MH716287.1	Vietnam	EV-A71 B5
MH716289.1	Vietnam	EV-A71 B5
MH716285.1	Vietnam	EV-A71 B5
MH716284.1	Vietnam	EV-A71 B5
MH716277.1	Vietnam	EV-A71 B5
MH716276.1	Vietnam	EV-A71 B5
MH716275.1	Vietnam	EV-A71 B5
MH716306.1	Vietnam	EV-A71 B5
MH716305.1	Vietnam	EV-A71 B5
MH716304.1	Vietnam	EV-A71 B5
MH716303.1	Vietnam	EV-A71 B5

Accession no.	Country	Virus
MH716302.1	Vietnam	EV-A71 B5
MH716379.1	Vietnam	EV-A71 B5
MH716260.1	Vietnam	EV-A71 B5
MH716295.1	Vietnam	EV-A71 B5
MH716294.1	Vietnam	EV-A71 B5
MH716297.1	Vietnam	EV-A71 B5
MH716296.1	Vietnam	EV-A71 B5
KJ686270.1	Vietnam	EV-A71 B5
KJ686211.1	Vietnam	EV-A71 B5
KJ686264.1	Vietnam	EV-A71 B5
KJ686222.1	Vietnam	EV-A71 B5
MH716372.1	Vietnam	EV-A71 B5
MH716261.1	Vietnam	EV-A71 B5
MH716369.1	Vietnam	EV-A71 B5
MH716374.1	Vietnam	EV-A71 B5
MH716309.1	Vietnam	EV-A71 B5
MH716317.1	Vietnam	EV-A71 B5
MH716316.1	Vietnam	EV-A71 B5
MH716314.1	Vietnam	EV-A71 B5
MH716315.1	Vietnam	EV-A71 B5
MH716318.1	Vietnam	EV-A71 B5
MH716313.1	Vietnam	EV-A71 B5
MH716312.1	Vietnam	EV-A71 B5
MH716301.1	Vietnam	EV-A71 B5
MH716308.1	Vietnam	EV-A71 B5
MH716320.1	Vietnam	EV-A71 B5
MH716368.1	Vietnam	EV-A71 B5
MH716307.1	Vietnam	EV-A71 B5
MH716370.1	Vietnam	EV-A71 B5
MH716378.1	Vietnam	EV-A71 B5
MH716377.1	Vietnam	EV-A71 B5
MH716376.1	Vietnam	EV-A71 B5
MH716373.1	Vietnam	EV-A71 B5
MH716371.1	Vietnam	EV-A71 B5
MH716332.1	Vietnam	EV-A71 B5
MH716333.1	Vietnam	EV-A71 B5
MH716334.1	Vietnam	EV-A71 B5
MH716329.1	Vietnam	EV-A71 B5
MH716327.1	Vietnam	EV-A71 B5
MH716324.1	Vietnam	EV-A71 B5
MH716328.1	Vietnam	EV-A71 B5
MH716326.1	Vietnam	EV-A71 B5
MH716325.1	Vietnam	EV-A71 B5
MH716331.1	Vietnam	EV-A71 B5
MH716330.1	Vietnam	EV-A71 B5
MH716335.1	Vietnam	EV-A71 B5
KJ686297.1	Vietnam	EV-A71 B5
KJ686234.1	Vietnam	EV-A71 B5
MH716323.1	Vietnam	EV-A71 B5
MH716344.1	Vietnam	EV-A71 B5
MH716342.1	Vietnam	EV-A71 B5
KJ686140.1	Vietnam	EV-A71 B5
KP308454.1	Cambodia	EV-A71 B5
KJ686302.1	Vietnam	EV-A71 B5
KJ686277.1	Vietnam	EV-A71 B5
KJ686296.1	Vietnam	EV-A71 B5
KJ686128.1	Vietnam	EV-A71 B5
MH716345.1	Vietnam	EV-A71 B5
KJ686192.1	Vietnam	EV-A71 B5
MH716338.1	Vietnam	EV-A71 B5
MH716322.1	Vietnam	EV-A71 B5
MH716321.1	Vietnam	EV-A71 B5
MH716346.1	Vietnam	EV-A71 B5
MH716337.1	Vietnam	EV-A71 B5
MH716336.1	Vietnam	EV-A71 B5
LC627081.1	Vietnam	EV-A71 B5
LC627079.1	Vietnam	EV-A71 B5
MH716298.1	Vietnam	EV-A71 B5
MH716300.1	Vietnam	EV-A71 B5

Accession no.	Country	Virus
MH716299.1	Vietnam	EV-A71 B5
MH716310.1	Vietnam	EV-A71 B5
MH716311.1	Vietnam	EV-A71 B5
MH716340.1	Vietnam	EV-A71 B5
MH716339.1	Vietnam	EV-A71 B5
MH716341.1	Vietnam	EV-A71 B5
MH716367.1	Vietnam	EV-A71 B5
MH716262.1	Vietnam	EV-A71 B5
MH716319.1	Vietnam	EV-A71 B5
MH716375.1	Vietnam	EV-A71 B5
LC627083.1	Vietnam	EV-A71 B5
LC627082.1	Vietnam	EV-A71 B5
LC627078.1	Vietnam	EV-A71 B5
LC627068.1	Vietnam	EV-A71 B5
MH716263.1	Vietnam	EV-A71 B5
MH716360.1	Vietnam	EV-A71 B5
MH716363.1	Vietnam	EV-A71 B5
MH716362.1	Vietnam	EV-A71 B5
MH716361.1	Vietnam	EV-A71 B5
MH716364.1	Vietnam	EV-A71 B5
MH716354.1	Vietnam	EV-A71 B5
MH716356.1	Vietnam	EV-A71 B5
MH716355.1	Vietnam	EV-A71 B5
MH716358.1	Vietnam	EV-A71 B5
MH716357.1	Vietnam	EV-A71 B5
MH716353.1	Vietnam	EV-A71 B5
MH716351.1	Vietnam	EV-A71 B5
MH716350.1	Vietnam	EV-A71 B5
MH716349.1	Vietnam	EV-A71 B5
MH716352.1	Vietnam	EV-A71 B5
MH716348.1	Vietnam	EV-A71 B5
MH716347.1	Vietnam	EV-A71 B5
MH716365.1	Vietnam	EV-A71 B5
MK344771.1	China	EV-A71 B5
LC627084.1	Vietnam	EV-A71 B5
MN966512.1	China	EV-A71 B5
LC627067.1	Vietnam	EV-A71 B5
LC627075.1	Vietnam	EV-A71 B5
LC627076.1	Vietnam	EV-A71 B5
LC627071.1	Vietnam	EV-A71 B5
LC627077.1	Vietnam	EV-A71 B5
LC627073.1	Vietnam	EV-A71 B5
LC627074.1	Vietnam	EV-A71 B5
LC627072.1	Vietnam	EV-A71 B5
LC627080.1	Vietnam	EV-A71 B5
MH716359.1	Vietnam	EV-A71 B5
KU647000.1	China	EV-A71 B5
KX372321.1	Thailand	EV-A71 B5
KX372320.1	Thailand	EV-A71 B5
LC375766.1	Japan	EV-A71 B5
FJ357378.1	Taiwan	EV-A71 B5
MN053435.1	Malaysia	EV-A71 B5
MN053432.1	Malaysia	EV-A71 B5
MN053433.1	Malaysia	EV-A71 B5
MN053431.1	Malaysia	EV-A71 B5
MN053430.1	Malaysia	EV-A71 B5
MN966518.1	China	EV-A71 B5
MN966517.1	China	EV-A71 B5
MN966516.1	China	EV-A71 B5
MN966515.1	China	EV-A71 B5
MN966514.1	China	EV-A71 B5
MN966513.1	China	EV-A71 B5
KY952186.1	Thailand	EV-A71 B5
KX372312.1	Thailand	EV-A71 B5
KX372313.1	Thailand	EV-A71 B5
KU574619.1	Thailand	EV-A71 B5
KP308430.1	Cambodia	EV-A71 B5
KP308448.1	Cambodia	EV-A71 B5
KX372311.1	Thailand	EV-A71 B5

Accession no.	Country	Virus
KX372310.1	Thailand	EV-A71 B5
JF738001.1	Thailand	EV-A71 B5
KX372317.1	Thailand	EV-A71 B5
KX372318.1	Thailand	EV-A71 B5
KR045296.1	Thailand	EV-A71 B5
KX372319.1	Thailand	EV-A71 B5
KR045304.1	Thailand	EV-A71 B5
KR045291.1	Thailand	EV-A71 B5
KR045293.1	Thailand	EV-A71 B5
KR045294.1	Thailand	EV-A71 B5
KR045295.1	Thailand	EV-A71 B5
KR045297.1	Thailand	EV-A71 B5
LC321989.1	Japan	EV-A71 B5
LC321993.1	Japan	EV-A71 B5
LC321992.1	Japan	EV-A71 B5
MH716380.1	Vietnam	EV-A71 B5
KX372322.1	Thailand	EV-A71 B5
KU888089.1	Vietnam	EV-A71 C1
KU888092.1	Vietnam	EV-A71 C1
JQ766160.1	China	EV-A71 C1
HQ285092.1	Singapore	EV-A71 C1
HQ676174.1	Finland	EV-A71 C1
HQ285091.1	Singapore	EV-A71 C1
KJ407272.1	Peru	EV-A71 C1
KJ407271.1	Peru	EV-A71 C1
JQ766161.1	China	EV-A71 C1
OP672344.1	Netherlands	EV-A71 C1
OP672343.1	Netherlands	EV-A71 C1
OP672363.1	Netherlands	EV-A71 C1
OP672349.1	Netherlands	EV-A71 C1
OP672346.1	Netherlands	EV-A71 C1
OP672351.1	Netherlands	EV-A71 C1
OP672348.1	Netherlands	EV-A71 C1
OP672362.1	Netherlands	EV-A71 C1
OP672350.1	Netherlands	EV-A71 C1
OP672361.1	Netherlands	EV-A71 C1
OP672358.1	Netherlands	EV-A71 C1
OP672353.1	Netherlands	EV-A71 C1
OP672354.1	Netherlands	EV-A71 C1
OP672347.1	Netherlands	EV-A71 C1
OP672352.1	Netherlands	EV-A71 C1
KU641489.1	Germany	EV-A71 C1
KU641488.1	Germany	EV-A71 C1
KU641487.1	Germany	EV-A71 C1
KF906434.1	India	EV-A71 C1
MK652139.1	USA	EV-A71 C1
KF906433.1	India	EV-A71 C1
KF906432.1	India	EV-A71 C1
KF906428.1	India	EV-A71 C1
KF906427.1	India	EV-A71 C1
KF906431.1	India	EV-A71 C1
KF906430.1	India	EV-A71 C1
KF906429.1	India	EV-A71 C1
MT641405.1	United Kingdom	EV-A71 C1
MG367608.1	Denmark	EV-A71 C1
MF770700.1	France	EV-A71 C1
MG367607.1	Denmark	EV-A71 C1
MG367604.1	Denmark	EV-A71 C1
MF770660.1	France	EV-A71 C1
MK111397.1	Cyprus	EV-A71 C1
MK111396.1	Cyprus	EV-A71 C1
KY865899.1	Netherlands	EV-A71 C1
MW731999.1	Germany	EV-A71 C1
MG367605.1	Denmark	EV-A71 C1
MG604318.1	Greece	EV-A71 C1
MG604317.1	Greece	EV-A71 C1
KY796193.1	India	EV-A71 C1
MG367600.1	Denmark	EV-A71 C1
LR027539.1	France	EV-A71 C1

Accession no.	Country	Virus
KX139462.1	Germany	EV-A71 C1
KU641502.1	Germany	EV-A71 C1
KU641495.1	Germany	EV-A71 C1
KU641503.1	Germany	EV-A71 C1
KU641507.1	Germany	EV-A71 C1
KU641504.1	Germany	EV-A71 C1
MG367596.1	Denmark	EV-A71 C1
MG367599.1	Denmark	EV-A71 C1
KU641494.1	Germany	EV-A71 C1
KU641493.1	Germany	EV-A71 C1
MH472687.1	Germany	EV-A71 C1
KU641505.1	Germany	EV-A71 C1
MH472686.1	Germany	EV-A71 C1
KU641497.1	Germany	EV-A71 C1
MH472702.1	Germany	EV-A71 C1
KU641496.1	Germany	EV-A71 C1
KU641492.1	Germany	EV-A71 C1
MH472707.1	Germany	EV-A71 C1
MH472705.1	Germany	EV-A71 C1
KU641498.1	Germany	EV-A71 C1
MG367598.1	Denmark	EV-A71 C1
KU641490.1	Germany	EV-A71 C1
MH472709.1	Germany	EV-A71 C1
MH472690.1	Germany	EV-A71 C1
MH472708.1	Germany	EV-A71 C1
MH472701.1	Germany	EV-A71 C1
MH472688.1	Germany	EV-A71 C1
MH472717.1	Germany	EV-A71 C1
KU641508.1	Germany	EV-A71 C1
MH472703.1	Germany	EV-A71 C1
MH472673.1	Germany	EV-A71 C1
MH472689.1	Germany	EV-A71 C1
MH472672.1	Germany	EV-A71 C1
MH472671.1	Germany	EV-A71 C1
MH472670.1	Germany	EV-A71 C1
MH410286.1	Poland	EV-A71 C1
KY991470.1	Poland	EV-A71 C1
MH472722.1	Germany	EV-A71 C1
MH472720.1	Germany	EV-A71 C1
MH472706.1	Germany	EV-A71 C1
LR027546.1	France	EV-A71 C1
LR027524.1	France	EV-A71 C1
MN397864.1	Germany	EV-A71 C1
MN397862.1	Germany	EV-A71 C1
MH472716.1	Germany	EV-A71 C1
MH472725.1	Germany	EV-A71 C1
LR027528.1	France	EV-A71 C1
KU641501.1	Germany	EV-A71 C1
LR027527.1	France	EV-A71 C1
MH472726.1	Germany	EV-A71 C1
MH472719.1	Germany	EV-A71 C1
MH472718.1	Germany	EV-A71 C1
MN397873.1	Germany	EV-A71 C1
MN397885.1	Germany	EV-A71 C1
MN397853.1	Germany	EV-A71 C1
MN397877.1	Germany	EV-A71 C1
MT081373.1	USA	EV-A71 C1
MT081374.1	USA	EV-A71 C1
MK800119.1	USA	EV-A71 C1
MW354746.1	Thailand	EV-A71 C1
MW354745.1	Thailand	EV-A71 C1
MW354744.1	Thailand	EV-A71 C1
MW354743.1	Thailand	EV-A71 C1
MW354742.1	Thailand	EV-A71 C1
MW354741.1	Thailand	EV-A71 C1
MW354740.1	Thailand	EV-A71 C1
MW354739.1	Thailand	EV-A71 C1
MN397905.1	Germany	EV-A71 C1
MN397887.1	Germany	EV-A71 C1

Accession no.	Country	Virus
MN397889.1	Germany	EV-A71 C1
MN397867.1	Germany	EV-A71 C1
MN397897.1	Germany	EV-A71 C1
MH410289.1	Poland	EV-A71 C1
MH410288.1	Poland	EV-A71 C1
MW354738.1	Thailand	EV-A71 C1
MN397890.1	Germany	EV-A71 C1
MW132449.1	Russia	EV-A71 C1
MN397879.1	Germany	EV-A71 C1
KY888026.1	USA	EV-A71 C1
MT747898.1	Taiwan	EV-A71 C1
MT747896.1	Taiwan	EV-A71 C1
MT747894.1	Taiwan	EV-A71 C1
MT747897.1	Taiwan	EV-A71 C1
MT747892.1	Taiwan	EV-A71 C1
MT747895.1	Taiwan	EV-A71 C1
MT747891.1	Taiwan	EV-A71 C1
MH256663.1	Switzerland	EV-A71 C1
MH256662.1	Switzerland	EV-A71 C1
MW132453.1	Russia	EV-A71 C1
MW132443.1	Russia	EV-A71 C1
MW132452.1	Russia	EV-A71 C1
MW132447.1	Russia	EV-A71 C1
MW132445.1	Russia	EV-A71 C1
MW132444.1	Russia	EV-A71 C1
MW132442.1	Russia	EV-A71 C1
MW132448.1	Russia	EV-A71 C1
MW132451.1	Russia	EV-A71 C1
MN397902.1	Germany	EV-A71 C1
MN397901.1	Germany	EV-A71 C1
MN397893.1	Germany	EV-A71 C1
MW132446.1	Russia	EV-A71 C1
MN397898.1	Germany	EV-A71 C1
LR027525.1	France	EV-A71 C1
MH472728.1	Germany	EV-A71 C1
MH472727.1	Germany	EV-A71 C1
MN397839.1	Germany	EV-A71 C1
MW132450.1	Russia	EV-A71 C1
MN397856.1	Germany	EV-A71 C1
MH472729.1	Germany	EV-A71 C1
LR027533.1	France	EV-A71 C1
MG367595.1	Denmark	EV-A71 C1
MG367597.1	Denmark	EV-A71 C1
MH256664.1	Switzerland	EV-A71 C1
MG367609.1	Denmark	EV-A71 C1
MF770701.1	France	EV-A71 C1
MF770698.1	France	EV-A71 C1
MF770671.1	France	EV-A71 C1
MF770668.1	France	EV-A71 C1
MF770688.1	France	EV-A71 C1
MF770665.1	France	EV-A71 C1
MF770663.1	France	EV-A71 C1
MF770669.1	France	EV-A71 C1
MF770682.1	France	EV-A71 C1
MF770662.1	France	EV-A71 C1
MF770695.1	France	EV-A71 C1
MF770696.1	France	EV-A71 C1
MG604327.1	Greece	EV-A71 C1
MG604325.1	Greece	EV-A71 C1
MG604326.1	Greece	EV-A71 C1
MG604324.1	Greece	EV-A71 C1
MG604321.1	Greece	EV-A71 C1
MG604320.1	Greece	EV-A71 C1
MW196699.1	Argentina	EV-A71 C1
MW732001.1	Germany	EV-A71 C1
MW732000.1	Germany	EV-A71 C1
MK836181.1	USA	EV-A71 C1
MK836180.1	USA	EV-A71 C1
MK836130.1	USA	EV-A71 C1

Accession no.	Country	Virus
MK836127.1	USA	EV-A71 C1
MK836112.1	USA	EV-A71 C1
MK836179.1	USA	EV-A71 C1
MK836170.1	USA	EV-A71 C1
MK836116.1	USA	EV-A71 C1
MK836123.1	USA	EV-A71 C1
MK836177.1	USA	EV-A71 C1
MK836163.1	USA	EV-A71 C1
MK836173.1	USA	EV-A71 C1
MK836164.1	USA	EV-A71 C1
OP762480.1	Russia	EV-A71 C1
MN397906.1	Germany	EV-A71 C1
MN397872.1	Germany	EV-A71 C1
MN397861.1	Germany	EV-A71 C1
MN397871.1	Germany	EV-A71 C1
OK605915.1	Brazil	EV-A71 C1
MK111399.1	Cyprus	EV-A71 C1
MK111401.1	Cyprus	EV-A71 C1
MK111400.1	Cyprus	EV-A71 C1
MK111404.1	Cyprus	EV-A71 C1
MK111403.1	Cyprus	EV-A71 C1
MK111402.1	Cyprus	EV-A71 C1
JQ315093.1	China	EV-A71 C4
MH395138.1	Spain	EV-A71 C1
MH395119.1	Spain	EV-A71 C1
MH394930.1	Spain	EV-A71 C1
MH395031.1	Spain	EV-A71 C1
MH395130.1	Spain	EV-A71 C1

Appendix Table 2. Genbank accession numbers and results for whole-genome sequencing of enterovirus A71 from Vietnam

Accession no.*	PCR cycle thresholds	Provinces/cities	Genome coverage (%)
OR766760	23.2	Ho Chi Minh City	99.8
OR766762	28.67	Binh Duong	98.9
OR766765	28.05	Tien Giang	99.7
OR766764	30.1	Binh Duong	99.1
OR766761	26.91	Ho Chi Minh City	99
OR766766	30	Tay Ninh	99.5
OR766763	26.8	Ho Chi Minh City	100
OR766768	25.77	Ho Chi Minh City	99
OR766770	27.86	Ho Chi Minh City	96.1
OR766769	29.6	Long An	98.5
OR766771	29.92	Ho Chi Minh City	96.9
OR766774	29.5	Dong Nai	95.9
OR791508	21.95	Hau Giang	98.9
OR791509	27.94	Dong Thap	96.9
OR791510	26.6	An Giang	98.2
OR822244	25.99	Ca Mau	86.3

*Genbank accession numbers for viral protein 1 gene sequences were OR766705–12, OR766716–8, OR766720–31, OR766732–8, and OR766740–58.

Appendix Table 3. Mean interlineage and intralineage sequence similarities for enterovirus A71 subgenogroup B5*

Lineage†	I	II	III	IV	V	VI	VII	VIII	IX	X
I	**99.18**	NA	NA	NA	NA	NA	NA	NA	NA	NA
II	95.58	**98.82**	NA	NA	NA	NA	NA	NA	NA	NA
III	96.05	96.01	**97.53**	NA	NA	NA	NA	NA	NA	NA
IV	95.84	95.72	96.63	**97.68**	NA	NA	NA	NA	NA	NA
V	96.68	95.99	96.91	96.76	**99.66**	NA	NA	NA	NA	NA
VI	95.53	95.19	95.80	95.36	96.40	**98.81**	NA	NA	NA	NA
VII	94.70	93.91	94.84	94.49	95.42	96.32	**98.92**	NA	NA	NA
VIII	92.93	92.60	92.97	93.27	94.09	94.32	93.73	**99.70**	NA	NA
IX	92.28	92.09	92.28	92.51	93.15	93.51	92.78	97.03	**99.62**	NA
X	93.82	93.70	94.23	94.19	94.89	95.61	95.55	94.84	93.84	**97.54**

*Numbers in bold font indicate intralineage sequence similarities. Interlineage sequences similarities were 92.09%–97.03%; intralineage sequence similarities were 97.53%–99.70%. NA, not applicable.
†Lineage placements within phylogenetic tree are shown in Figure 2 (main text) and Appendix Figure 6.

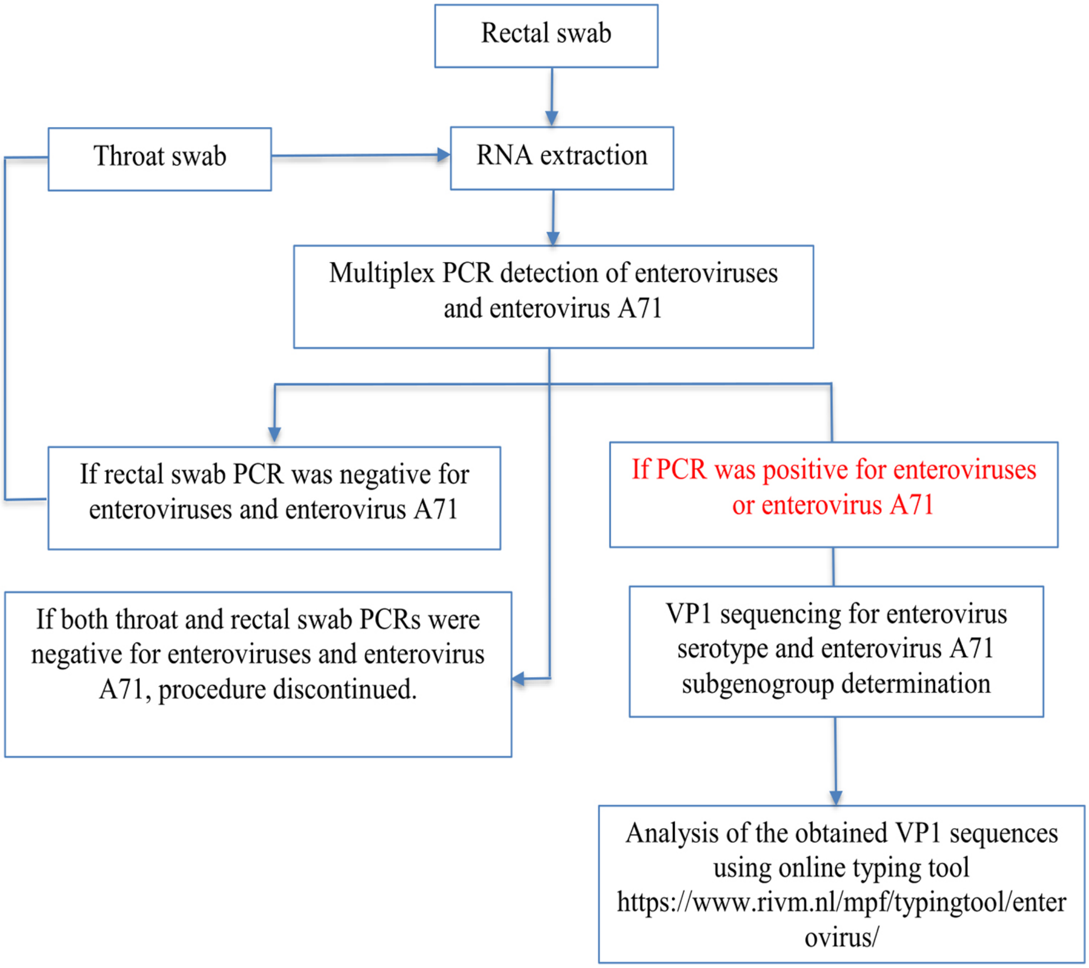

Appendix Figure 1. Flowchart showing the laboratory workflow used to diagnose enteroviruse and enterovirus A71 infections causing hand, foot, and mouth disease in Vietnam, 2023.

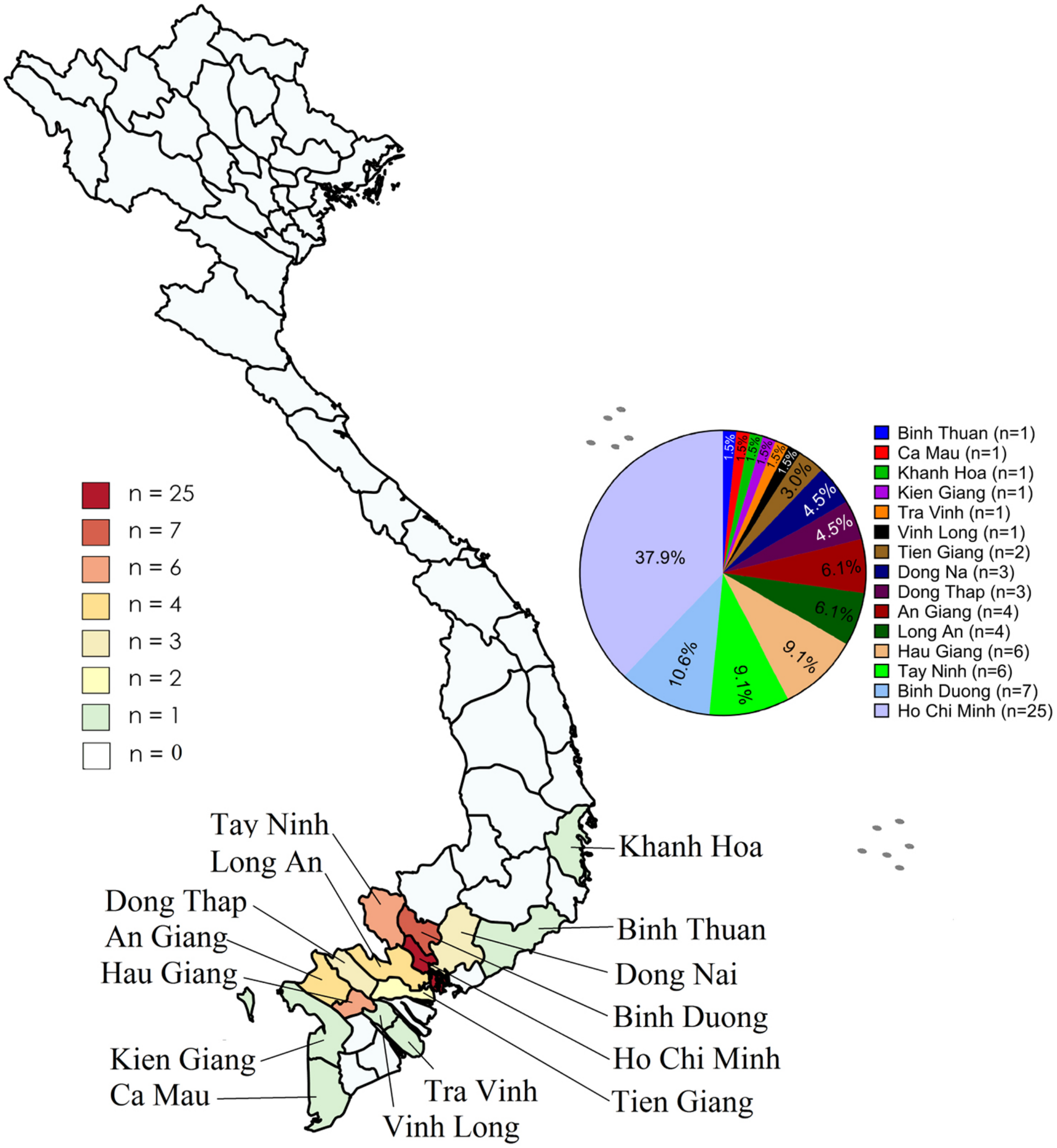

Appendix Figure 2. Map showing the geographic distribution of 101 patients who were enrolled in the clinical study of emerging enterovirus A71 subgenogroup B5 causing severe hand, foot, and mouth disease, Vietnam, 2023. Colored regions in the map indicate the number of patients from that region. Pie chart indicates the percentage of patients within each region.

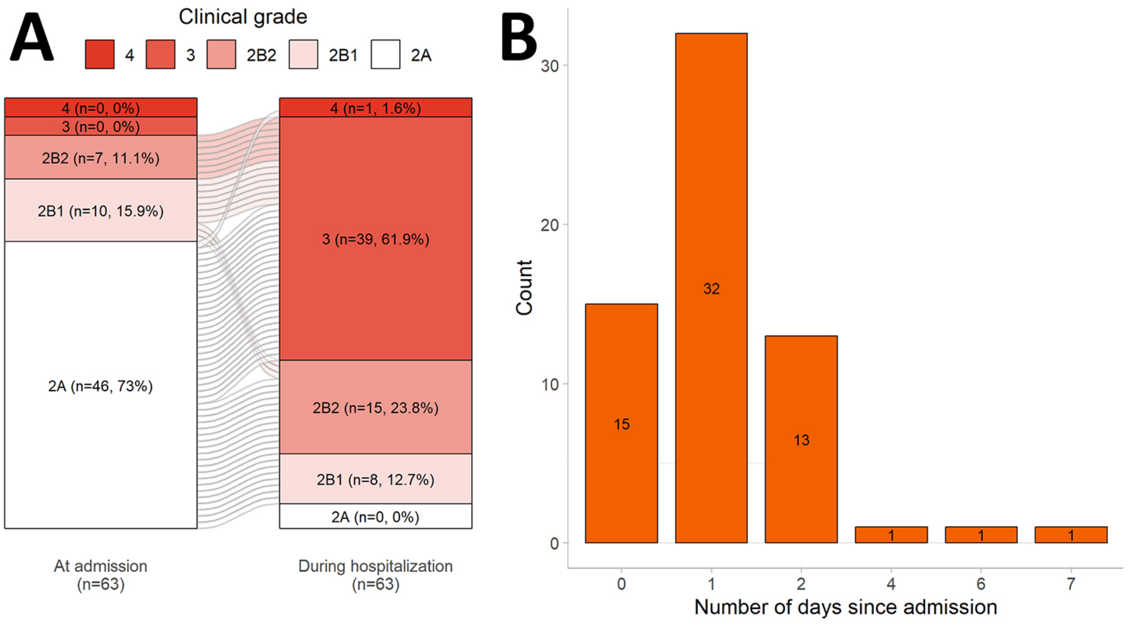

Appendix Figure 3. Clinical grade and progression of hand, foot, and mouth disease in patients from a 2023 outbreak in Vietnam. A) Trajectory of 63 of 101 hospitalized study participants who progressed from a lower to higher clinical severity grade of hand, foot, and mouth disease since admission. Clinical grades of hand, foot, and mouth disease have been previously defined (*3*, main text). B) Number of patients who progressed to higher clinical severity within 7 days after hospital admission (total number was 63). Numbers within bars indicate the number of patients who progressed at each time point.

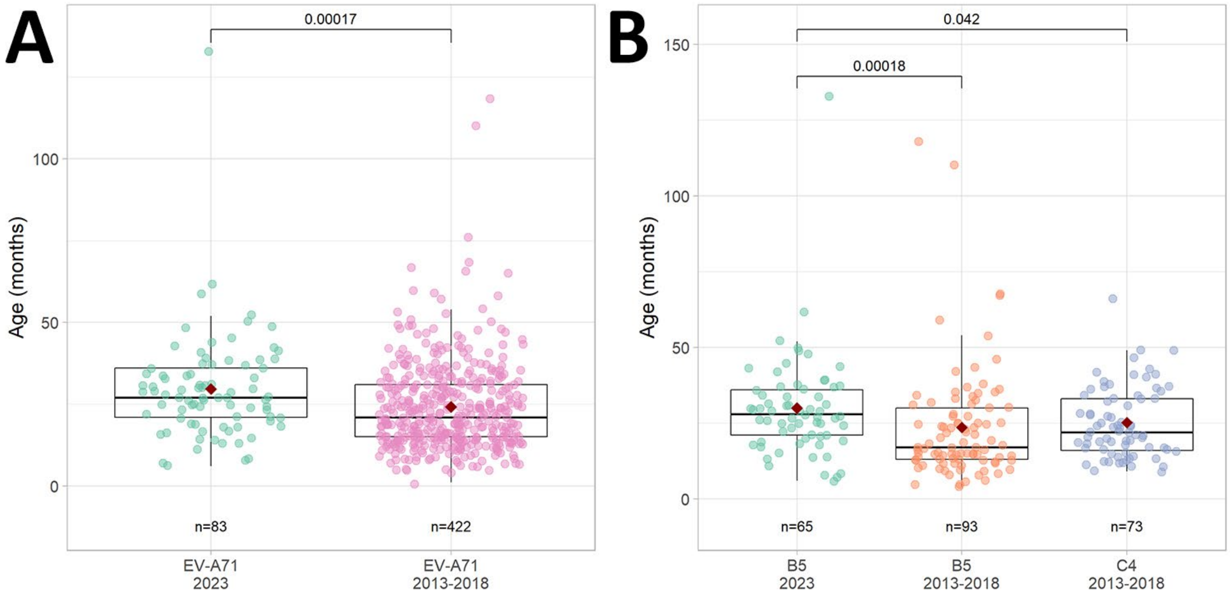

Appendix Figure 4. Boxplots showing the differences in ages among patient groups in study of emerging enterovirus A71 subgenogroup B5 causing severe hand, foot, and mouth disease, Vietnam, 2023. A) Comparison of ages for patients who had EV-A71 detected during 2023 vs. EV-A71 detected during

2013–2018. Median months of age (interquartile range) were 27 (21–36) for EV-A71 during 2023 and 21 (15–31) for EV-A71 detected during 2013–2018; p value is shown at the top of the graph. B) Comparison of ages for patients who were infected with different EV-A71 subgenogroups. Median months of age (interquartile range) were 28 (21–36) for EV-A71 B5 detected during 2023 (28:), 18 (13–30) for EV-A71 B5 detected during 2013–2018, and 22 (17–33) for EV-A71 C4 detected during 2013–2018; p values for each comparison are shown at the top. Wilcoxon rank-sum tests with continuity correction were applied for analyses of patient ages among groups. EV-A71, enterovirus A71.

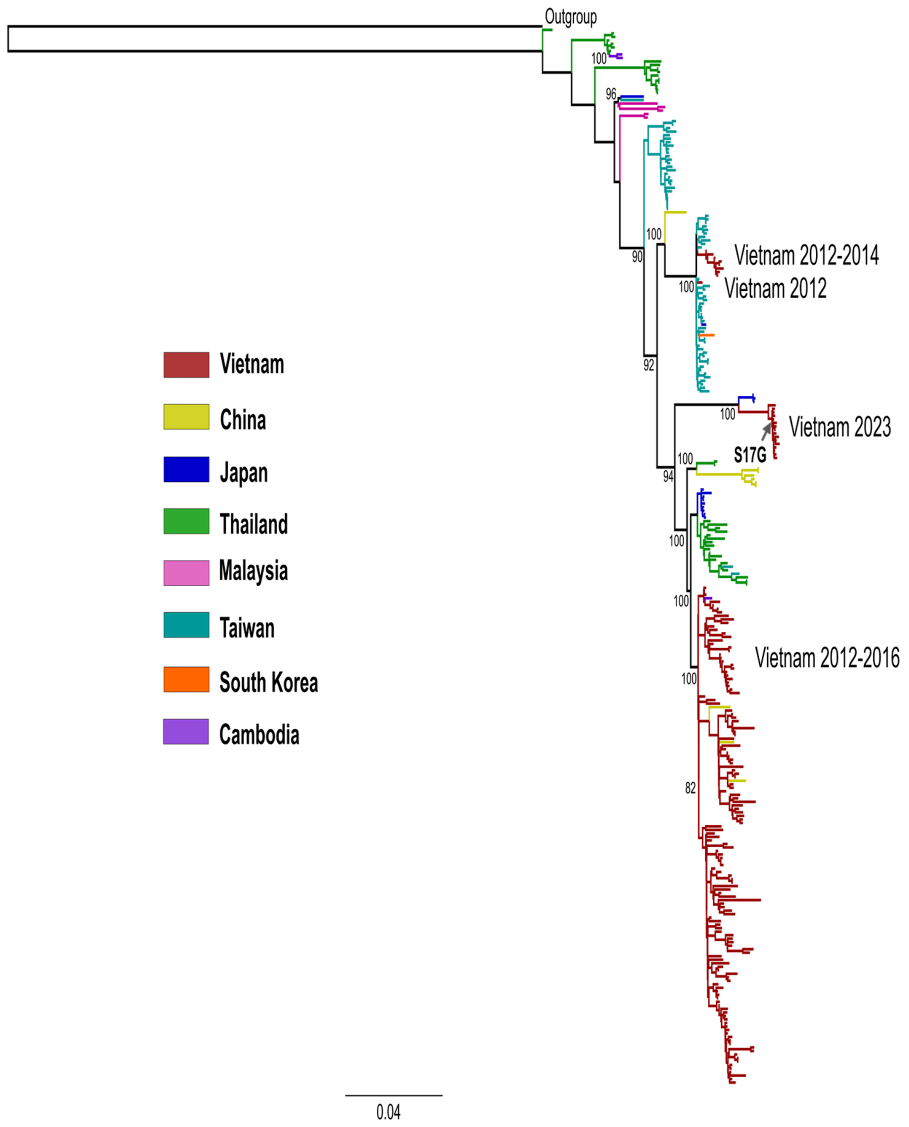

Appendix Figure 5. Phylogenetic analysis of complete coding sequences of emerging enterovirus A71 subgenogroup B5 causing severe hand, foot, and mouth disease, Vietnam, 2023. Tree was constructed by using the maximum-likelihood method to show genetic relatedness among the EVA-71 subgenogroup B5 obtained in this study compared with global sequences from GenBank. Colored lines in the tree indicate the country of origin for each sequence. Scale bar indicates nucleotide substitutions per site.

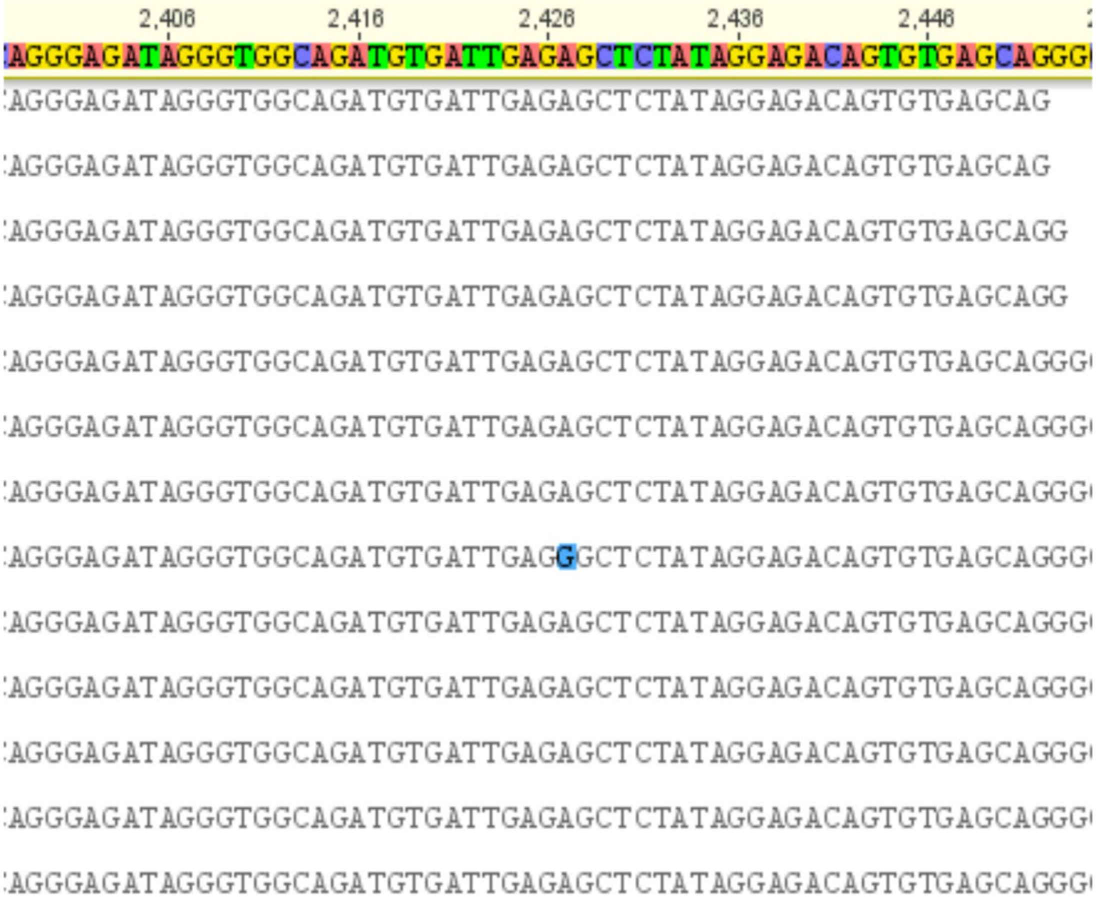

Appendix Figure 6. Screen shot of enterovirus viral protein 1 sequence showing a glycine (GGC) codon instead of serine (AGC) in study of emerging enterovirus A71 subgenogroup B5 causing severe hand, foot, and mouth disease, Vietnam, 2023. Blue shading indicates the position of the nucleotide change. Colored sequence at the top is the consensus sequence.S17G substitution was found in the N-terminus of the viral protein 1 protein in 15 of 16 B5 sequences from the 2023 outbreak in Vietnam.

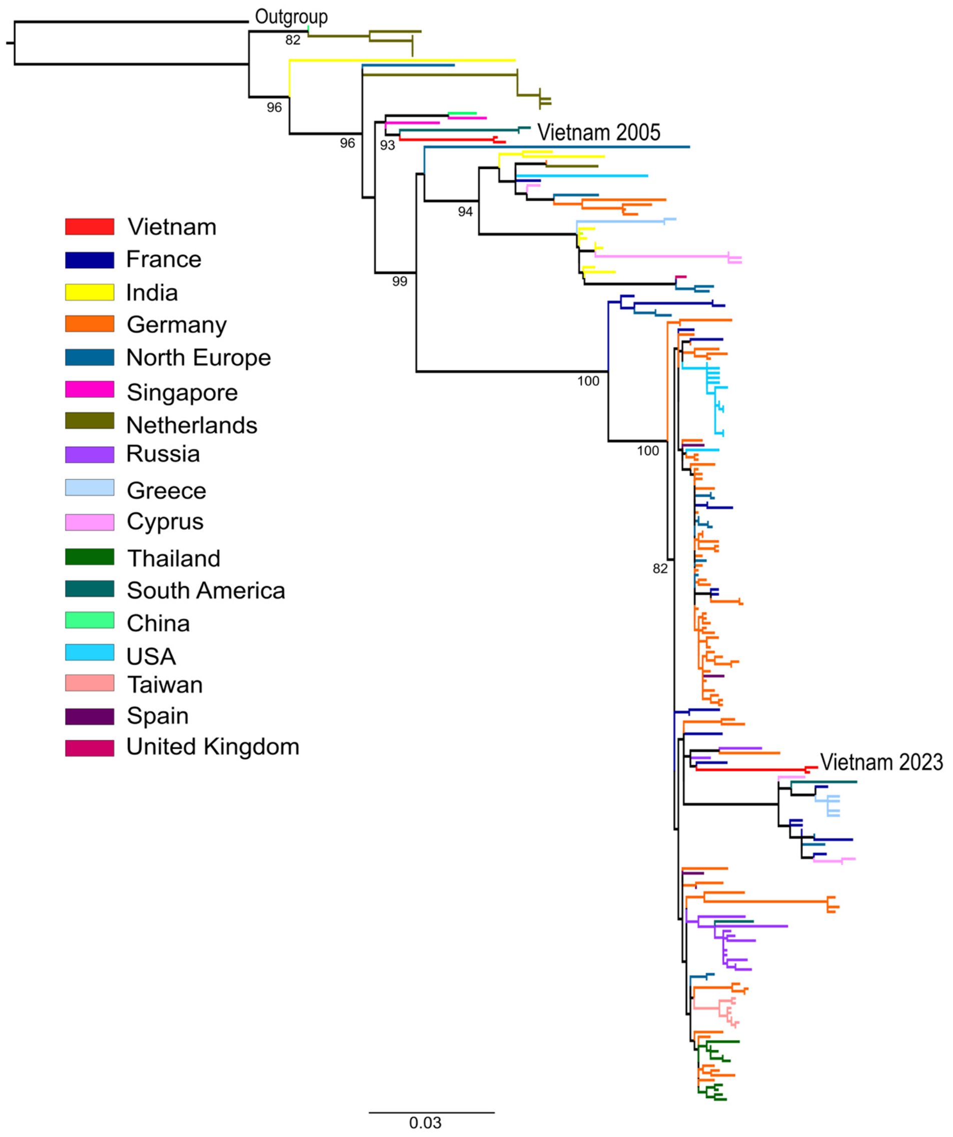

Appendix Figure 7. Phylogenetic analysis of viral protein 1 gene sequences from enterovirus A71 subgenogroup C1 identified in study of severe hand, foot, and mouth disease, Vietnam, 2023. Tree was constructed by using the maximum-likelihood method to show genetic relatedness among EV-A71 subgenogroup C1 sequences obtained in this study and global sequences obtained from GenBank. Colored lines indicate the country of origin for each sequence. Scale bar indicates nucleotide substitutions per site.
